# Outcomes of the Surgical Treatment of Periprosthetic Fractures Around the Knee with Locking Plates: A Single Centre Experience

**DOI:** 10.5704/MOJ.2111.001

**Published:** 2021-11

**Authors:** IB Atalay, R Ozturk, A Yapar, Y Karakoc, MF Eksioglu

**Affiliations:** Department of Orthopaedics and Traumatology, Dr Abdurrahman Yurtaslan Onkoloji Egitim ve Arastirma Hastanesi, Ankara, Turkey

**Keywords:** total knee arthroplasty, periprosthetic fracture, locking compression plates

## Abstract

**Introduction:**

Surgical treatment options for periprosthetic fractures (PPF) include internal fixation with plate, intramedullary nailing and revision arthroplasty. We aimed at evaluating the surgical outcomes of patients who we had treated PPF with locking compression plates (LCP).

**Materials and methods:**

Twenty patients with PPF after primary total knee arthroplasty (TKA) between 2009 and 2016 were included in to the study. Knee Society Knee Scoring System (KSKSS) was used in the evaluation of radiologic and functional outcomes. There were periprosthetic supracondylar femoral fractures in 15 patients, and that of tibial fractures in 5 patients. For internal fixation, locking compression plate was preferred.

**Results:**

The mean age was 69 (range 61 to 78) years and the mean follow-up period was 72.25 (range 24 to 110) months. Union was achieved by 15.8 weeks in all the cases. Superficial infection and implant fracture were each seen in two patients. Revision operations were done to those patients with implant fracture. Mean KSKSS was 81.4 (75-87) and the mean functional score was 78.75 (75-85). Degenerative osteoarthritis patients were found to have higher age values than post-traumatic osteoarthritis patients (p = 0.001). When the union times were compared, it was found that the degenerative osteoarthritis patient group had a significantly shorter union than the post-traumatic osteoarthritis patient group (p = 0.036).

**Conclusion:**

Internal fixation with LCP is an effective treatment method in managing of PPF for patients with good bone stock. Rigid fixation should be done with the right surgical technique and an early movement must be initiated so that a good function can be achieved.

## Introduction

In recent years, the numbers of arthroplasty implementations have been increasing in parallel to increasing life expectancy and the increasing quality of life. Therefore, PPF incidence is also increasing^1^.

The incidence of periprosthetic fractures (PPF) after total knee arthroplasty (TKA) is between 0.3% - 2.5%. The most common fracture after knee arthroplasty is supracondylar femoral fractures and they include fractures up to 15cm from the joint^2,3^. This is followed by patella fractures and proximal tibial fractures^4^.

Fracture may be seen intra-operatively or post-operatively. As early fractures generally seen from the surgical technique, late fractures are due to low energy traumas. Also, the factors leading to fracture can be classified as the ones related to patient or the ones related to the surgery. Risk factors related to patient are osteoporosis, rheumatoid arthritis, neurologic diseases, steroid usage, smoking, immunosuppression, and female sex. Surgical factors are excessive bone resection and anterior femoral notching^1,4^.

Although the intra-operative fractures are relatively less displaced, post-operative fractures are more fragmented fractures accompanied by soft tissue trauma. Also, the fixation of prosthesis may be affected. Arthroplasty patients must be mobilised early due to the fact that they are elderly patients with accompanying systemic diseases and their general medical conditons. Moreover, accompanying osteoporosis makes fixation difficult and mobilisation late. These factors complicate the management of PPF after arthroplasty.

## Materials and Methods

Between January 2009 and December 2016, 20 patients with informed consent who have PPF after primary TKA have been retrospectively evaluated. Sixteen of them were female and four of them were male. The time elapsed from the first surgery to the fracture, age, follow-up period, fracture localisation and the complications were evaluated. All patients were divided into two groups as patients between 60-69 years and patients aged 70 and over. Radiographic and functional outcomes evaluations are set according to Knee Society Knee Scoring System (KSKSS)^5^. According to this scoring system, in knee scoring; pain, flexion contracture (if present), extension range, total range of motion, varus-valgus angulations, and stability parameters were evaluated. On the other hand, in functional scoring, walking capacity, going up and down through stairs, walking apparatus usage were evaluated. According to these scoring; 60 points and below are considered as poor, 60-69 points as moderate, 70-79 points as good, 80-100 points are perfect.

**Fig 1:**
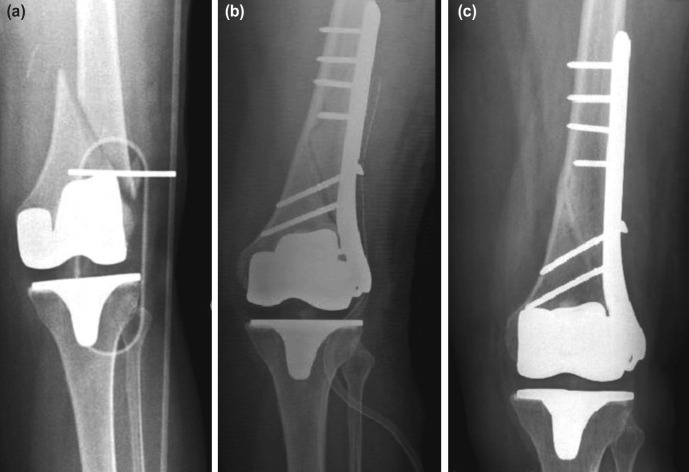
(a) Lewis Rorabeck Type 2 supracondylar femoral periprosthetic fracture. (b) Post-operative first day radiography. (c) 16th week radiography.

**Fig 2:**
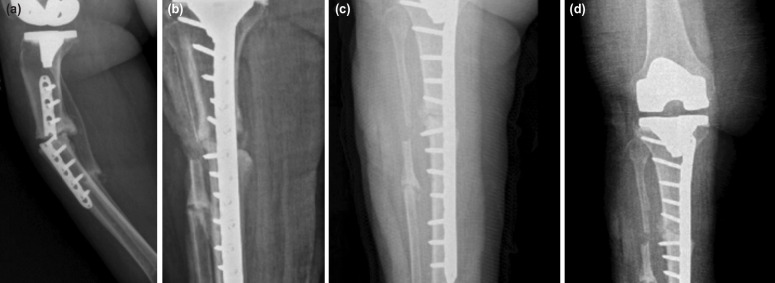
(a) Implant failure after operation for Felix type 3 periprosthetic fracture. (b) Internal fixation by means of iliacautogenous grafting and locking compression plate (c) 16th week radiography. (d) 24th week radiography.

For all our patients on first application, physical examination, and two-direction conventional direct graphs were applied. In 15 cases PPF was seen as supracondylar femoral fracture, on the other hand it was periprosthetic tibial fracture for 5 cases. Twelve of 15 supracondylar femoral fractures were Lewis and Rorabeck^6^ type 2 fractures, 3 was type 1 and internal fixation with Locking compression plate (LCP) with minimal invasive technique was done to all the fractures (Fig. 1). No prosthesis cut-off was seen after fracture. Tibial fractures were Felix type 3A fractures^7^. Due to the fact that prosthesis stability was strong, internal fixation with LCP with minimal invasive technique was applied for these fractures too (Fig. 2). Post-operative follow-ups were done monthly.

Statistical analysis was performed using the IBM SPSS version 22.0 software [IBM Corp, Armonk, NY, USA]. Categorical variables were expressed in number and percentage, while continuous variables were expressed in mean ± standard deviation (SD) and median (min-max) values. The relevance of continuous variables to normal distribution was evaluated using the visual (histogram and probability graphics) and analytical methods (Kolmogorov-Smirnov/Shapiro-Wilk tests). For categorical variables, whether there was a difference with respect to frequency between the groups was compared using the chi-square test. The Mann-Whitney U test was used for the comparison of abnormally distributed data between the groups. The Wilcoxon test was used to evaluate the change in pain score before and after treatment. A p value of <0.05 was considered statistically significant.

## Results

The mean age of patients was 69 years (range, 61-78 years) and the mean follow-up period was 72.3 months (range, 24-110 months). In none of the patients there had been a complication post-operatively and the fracture reason was simple fall in all of the cases. The time from TKA to the fracture was 45 months (range, 10-84 months). The indication of implementing TKA for 6 patients was posttraumatic osteoarthritis, and it was degenerative osteoarthritis for 14 patients. In two patients, superficial infection findings characterised by hyperemia over the skin and slight edema were observed in the follow-ups. No fistula or drainage was observed. Those patients were hospitalised and given parenteral antibiotic therapy. Symptoms of the two patients were totally alleviated. One of the patients operated for tibial fractures came back with implant insufficiency second month post-operatively and so came back the other one, third month post-operatively. For these two patients, iliac autogenous grafting and internal fixation was implemented. In all the patients union was obtained by 15.8 months (range, 12-24 months) months on average. The mean range of motion was 96.50° (range, 80° - 110°). KSKSS was 81.4 (range, 75-87) and the mean functional score was 78.75 (range, 75-85) (Table I).

**Table I:** Baseline characteristics

Parameters (n=20)	
Age, year	
Mean ± sd	69.0 ± 5.5
Median (min-max)	70.0 (61.0-78.0)
Gender, n (%)	
Male	4 (20.0)
Female	16 (80.0)
Diagnosis, n (%)	
Osteoarthritis	14 (70.0)
Post-traumatic osteoarthritis	6 (30.0)
PPF time, month	
Mean ± sd	45.0 ± 20.2
Median (min-max)	47.5 (10.0-84.0)
Treatment, (%)	
Open reduction, internal fixation	18 (90.0)
Open reduction, internal fixation + AutoGraft	2 (10.0)
Union time, week	
Mean ± sd	15.8 ± 2.6
Median (min-max)	15.5 (12.0-24.0)
Follow-up time, month	
Mean ± sd	72.3 ± 25.1
Median (min-max)	68.5 (24.0-110.0)
Knee score	
Mean ± sd	81.5 ± 3.8
Median (min-max)	80.0 (75.0-87.0)
Functional score	
Mean ± sd	78.8 ± 3.9
Median (min-max)	80.0 (75.0-85.0)
f-ROM, °	
Mean ± sd	96.5 ± 9.3
Median (min-max)	97.5 (80.0-110.0)

sd: standard deviation

PPF time: Time elapsed between the first fracture and the arthroplasty

f-ROM: Flexion range of motion

**Table II:** Spearman’s Correlation Coefficient between Age, Knee score, Functional score, Flexion range of motion degrees, PPF time and Union time

n=20	Union time	
	r	p
Age	-0.340	0.142
Knee score	-0.366	0.113
Functional score	-0.187	0.429
Flexion range of motion degree	-0.374	0.104
PPF time	-0.129	0.589

r: Spearman’s correlation coefficient

PPF time: Time elapsed between the first fracture and the arthroplasty

**Fig 3:**
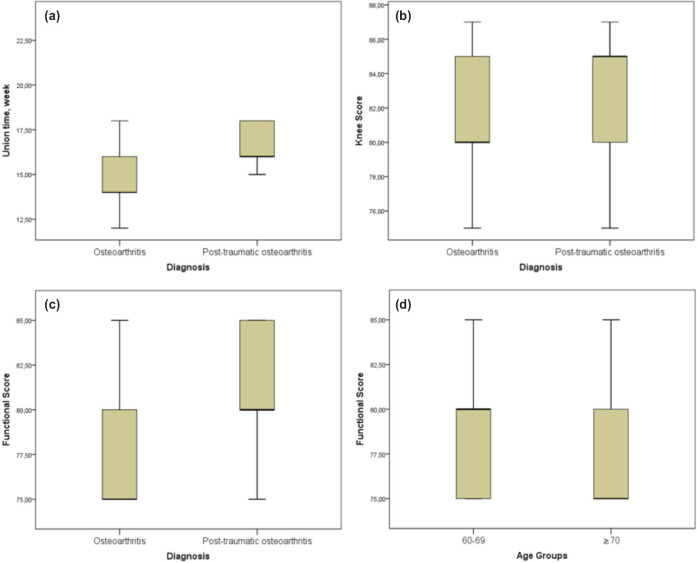
(a) Union time in diagnosis groups. (b) Knee score in diagnosis groups. (c) Functional scores in diagnosis groups. (d) Functional scores in age groups.

There was no statistically significant relationship between union time, age, knee score, functional score, flexion range of motion degree and PPF-time (p> 0.05) (Table II). The results of the comparison analyses between patient groups by diagnosis and age are presented in Table III. Degenerative osteoarthritis patients were found to have higher age values than posttraumatic osteoarthritis patients (p < 0.001).There was no significant difference between the sex distributions of both diagnostic groups and age groups (p = 0.549, p = 0.285, respectively). When the union times were compared, it was found that the degenerative osteoarthritis patients group had a significantly shorter union time than the posttraumatic osteoarthritis patients group (p = 0.036). Union time was similar in both age groups (p = 0.194) (Table III). Knee score (p=0.253 and p=0.571) and functional score (p=0.109 and p=0.436) was found to be similar between diagnosis groups and age groups (Fig. 3).

**Table III:** Spearman’s Correlation Coefficient between Age, Knee score, Functional score, Flexion range of motion degrees, PPF time and Union time

N=20	Diagnosis		P
	OA (n=14)	PTOA (n=6)	
Age, year			0.0011
Mean ± sd	71.8 ± 4.0	62.5 ± 1.0	
Median (min-max)	72.0 (65.0-78.0)	62.5 (61.0-64.0)	
Gender, n (%)			0.5492
Male	2 (14.3)	2 (33.3)	
Female	12 (85.7)	4 (66.7)	
PPF time, month			0.8041
Mean ± sd	44.5 ± 17.6	46.2 ± 27.2	
Median (min-max)	47.5 (10.0-84.0)	47.0 (11.0-80.0)	
Knee score			0.2531
Mean ± sd	80.9 ± 3.5	82.8 ± 4.5	
Median (min-max)	80.0 (75.0-87.0)	85.0 (75.0-87.0)	
Functional score			0.1091
Mean ± sd	77.8 ± 3.8	80.8 ± 3.8	
Median (min-max)	75.0 (75.0-85.0)	80.0 (75.0-85.0)	
f-ROM, °			0.4231
Mean ± sd	95.4 ± 9.1	99.2 ± 10.2	
Median (min-max)	95.0 (80.0-110.0)	100.0 (85.0-110.0)	
Union time, week			0.0361
Mean ± sd	15.1 ± 2.0	17.5 ± 3.3	
Median (min-max)	14.0 (12.0-20.0)	16.0 (15.0-24.0)	
Follow-up time, month			0.6801
Mean ± sd	73.4 ± 24.0	69.5 ± 29.7	
Median (min-max)	68.5 (24.0-110.0)	71.5 (24.0-110.0)	
	**Age**		
	**60-69 Ages (n=9)**	**≥70 (n=11)**	
Gender, n (%)			0.2852
Male	6 (66.7)	10 (90.0)	
Female	3 (33.3)	1 (9.1)	
PPF time, month			0.6481
Mean ± sd	47.7 ± 26.9	42.8 ± 13.6	
Median (min-max)	48.0 (11.0-84.0)	47.0 (10.0-61.0)	
Knee score			0.5711
Mean ± sd	81.9 ± 4.6	81.1 ± 3.3	
Median (min-max)	85.0 (75.0-87.0)	80.0 (75.0-87.0)	
Functional score			0.4361
Mean ± sd	79.4 ± 3.9	78.2 ± 4.0	
Median (min-max)	80.0 (75.0-85.0)	75.0 (75.0-85.0)	
f-ROM, °			0.6411
Mean ± sd	95.6 ± 10.4	97.3 ± 8.8	
Median (min-max)	95.0 (80.0-110.0)	100.0 (80.0-110.0)	
Union time, week			0.1941
Mean ± sd	16.6 ± 3.0	15.2 ± 2.2	
Median (min-max)	16.0 (14.0-24.0)	14.0 (12.0-20.0)	
Follow-up time, month			0.4701
Mean ± sd	76.9 ± 28.0	68.5 ± 23.1	
Median (min-max)	88.0 (36.0-108.0)	65.0 (24.0-110.0)	

^1^Mann-Whitney U test

^2^Chi-Square Test

OA: Osteoarthritis PTOA: Post-traumatic osteoarthritis

## Discussion

Treatment of periprosthetic fractures around the knee is challenging because of the complex fracture morphology, high rates of injury associated with osteopenia, and variability of injury patterns^4^. In this study, we aimed to investigate the results of surgical treatment of periprosthetic fractures of the knee with locking plates. Similar to the literature, this method can be applied in periprosthetic fractures and the results are good. Considering factors such as periprosthetic fractures around the knee, fracture site, type of patient age, they are most often treated with a locked plate or retrograde intramedullary nail (some distal femoral periprosthetic fractures^4,8-12^. These methods are superior to cast immobilisation, external fixation, dynamic compression plate fixation, and dynamic condylar screw fixation.

We preferred a locking plate instead of a nail for periprosthetic fractures around the knee. Because the bone quality of the patients was good and the fractures were displaced. In addition, the application of intramedullary nails is difficult because of the narrowed or closed medullary area due to total knee replacement. Because the notch should be extended frequently in nail application and this leads to concerns about early prosthesis loosening^11,13^. Furthermore, the locked compression plate is inserted through a small incision using a minimally invasive technique, thereby minimising damage to the periosteal blood supply and improving healing. As the plate is pre-shaped, it helps to reduce fracture fragments. Multiple screws can be placed at different angles, helping to prevent displacement and varus collapse of fracture fragments^8,14^. For two patients of ours that revised with more taller locking plates due to plate insufficiency we had also implemented iliac autogenous grafting due to insufficient bone reserve and obtained union in 20th and 24th weeks in order.

Traditional plate fixations are prone to varus collapse^15^. In the literature it has been reported that non-union rates are high with conventional plates. Figgie *et al*^16^ have reported in their 10-patient series that non-union rate was 50% with traditional plates. Ricci *et al*^9^ 24-patient series of supracondylar femoral fractures have reported non-union rate 86%. Fixed-angle blade plates or 95º condylar plates can prevent varus collapse, but are difficult to be placed in the presence of a prosthesis^16^.

Kregor *et al* implement locking plate to 103 patients for distal femoral fracture and they observed non-union requiring graft in only one patient^10^. Agarwal *et al*^8^ in their 20-patient series have implemented LCP for 11 patients and reported 100% union rate. We also implemented internal fixation with locking plates for 15 supracondylar femoral and 5 tibial PPF, and have obtained union in all patients. In our results, we did not encounter any complication such as varus collapse.

There are also articles in the literature that report high rates of complications after treatment of periprosthetic fractures with locking plates^8,12^. Non-union, delayed union, deep infection and implant failure have been reported. In our study, we did not see any deep infection. We detected superficial infection in two patients that we could treat with antibiotic therapy. We assigned implant failure in two patients and in these cases, we performed iliac autogenous grafting and revision with longer plates. We achieved complete union in these two patients.

Periprosthetic supracondylar femoral fractures are frequent in patients over 60 years old or patients with osteoporosis. Although they are generally seen after low energy traumas, traffic accidents and rehabilitation manoeuvres after arthroplasty are also risk factors for supracondylar femoral fractures^17^. It has been reported in the biomechanical studies concerning with anterior femoral notching that notching that is deeper that 3mm and around the end point of prosthesis leads to high level of stress and increases the risk of fracture^18^.

In the supracondylar femoral fracture, Lewis and Rorabeck classification is used. According to this classification, fractures that are non-displaced and without cut-off are type 1, displaced but without cut-off are type 2, displaced or/nondisplaced with cut-off fractures are type 3. Six of our patients were type 2 and one was type 1. Periprosthetic tibial fractures are less common. It is seen especially after revision knee arthroplasty and the most important reasons are trauma and component and joint instability with mal-alignment^1^. Tibial PPFs were evaluated according to Felix Classification, and according to this classification, fractures are divided into four. Type 1 is tibial plateau fracture, type 2 is metaphysial region fractures around lower point of tibial stem, type 3 is the fractures from the tibial stem to distal, and type 4 is tibial tubercle fractures. Fractures with intact prosthesis are type A, fractures with cut-off is type B, and intra-operative fractures are type C^19^.

In our series, the fractures are classified as Felix type 3A in tibial fractures. Twelve of 15 supracondylar femur fractures were Lewis - Rorabeck type 2 and 3 was type 1 fractures.

The goals of treatment in periprosthetic fracture surgery are to achieve a painless and stable knee joint, thus achieving painless and stable knees with excellent fit and range of motion. This ensures a good range of motion^8,20,21^. Conservative treatments with brace or cast result in reduced range of motion. Early return to the previous functional state can be achieved after the locking is secured by a good alignment with the plates. Agarwal *et al*^8^ reported the mean Knee Society Knee Score was 85 (range, 75-89) and the functional score was 76 (range, 70-85) in their patients. In this current study we found Knee Society Knee Score was 81.4 (range, 75-87) and the mean functional score was 78 .75 (range, 75-85).

There were some limitations of this study. Firstly, it was a retrospective analysis and the number of patients was relatively small, as it included the results of a single centre and was a rare condition. In addition, patella fractures were excluded because only periprosthetic fractures with locking plates were examined. Prospective, comparative studies with more patients are needed in the future.

## Conclusion

Post-operative PPF are seen as more complicated than intra-operative fractures. The aim of the revision surgery is early mobilisation and stable fixation. We have been assuming that internal fixation with LCP is a reliable treatment option for PPF with good bone reserve and stable prosthesis. In addition, the duration of union after periprosthetic fracture surgery is shorter in the degenerative osteoarthritis group compared to post-traumatic arthritis.
